# Current News and Opinion

**Published:** 1882-01

**Authors:** 


### CURRENT NEWS AND OPINION.

TYPHOID FEVER is said to prevail extensively in Toronto, Can.

MANIACAL INSANITY sometimes follows the excessive use of the bromides.

PROF. KLEBS has discovered a new bacillus, this time in typhoid fever. Next!

THE SICKNESS among the laborers on the Isthmus canal, is reported to be very much exaggerated.

SMALL-POX is so prevalent in Biddeford, Me., that all the public schools have been closed, in order to limit the spread of the epidemic.

DR. T. DAVIS FITCH, of Chicago, who has been recently seriously ill, has quite recovered, and resumed his practice.

DR. EMILY F. POPE, of Boston, has been elected Secretary of the American Social Science Association, in place of Mr. Frank B. Sanborn.

THE CHICAGO MEDICAL REVIEW, is authority for the statement, that a physician of that city recently mistook a case of confluent small-pox for typhoid fever.

BUT ONE CASE of hydrophobia was discovered among forty-seven thousand dogs, captured and disposed of in this city, during the past two years.

ELEVEN THOUSAND DOLLARS have been raised by subscription, towards a new building for the Boston Dispensary.

THE DEATH-RATE of Paris is only 20 per thousand, against 22 per thousand for London, 24 for Berlin, 27 for New York and 30 for Vienna.

DR. A. J. ROE, says he treats gonorrhœa with injections of boracic acid, ten grains to the ounce of water, night and morning, and cures his cases within ten days.

DR. JOHN S. BILLINGS, of the Army and Prof. H. J. Bigelow, of Boston, have been elected to honorary membership in the Clinical Society of London.

VIRCHOW recently celebrated the 25th anniversary of his appointment to a professorship in the University of Berlin. He has also been re-elected to the German Parliament.

A CASE OF SERIOUS POISONING in a school, from eating colored crayons, is reported by Dr. Cameron, in the *Dublin Journal of Medical Science.* The crayons were colored with arsenite of copper.

THE CHICAGO MEDICAL AUTHORITIES state that persons come to the Health office every day affected with small-pox and apply for admission to the pest house.

DURING THE VERY WARM WEATHER in August and September the Sanitary Association of New Orleans, caused the streets to be flushed with 5,000,000 to 6,000,000 gallons of water daily.

DRS. J. R. CHADWICK, E. H. Bradford and T. B. Curtis, were recently appointed instructors in the Medical Department of Harvard University. Dr. Curtis has since died.

PROF. BUSCH, of Bonn, has received the Grand Cross of the House of Hohenzollern, in recognition of the successful performance of an operation on the German Empress.

DR. E. M. HARTWELL says that the first recorded *post mortem* examination made in America, was done August 8th, 1670. Its record is found in a manuscript order of the Council of Lord Baltimore, dated at St. Mary's, in Maryland.

BARON JAMES DE ROTHSCHILD, died recently from a rupture of an aneurism resulting from mental anxiety, consequent on losses on the Paris Bourse. So says Mr. Henri Labouchere, in the *London Truth*.

MR. ERASMUS WILSON, who is distinguished alike as a scientific physician and a philanthropist, has been knighted by Queen Victoria for his many noble benefactions, and generous encouragement of medical study. The honor was worthily bestowed.

THE CHICAGO COLLEGE OF PHYSICIANS AND SURGEONS is a new institution in that city. Professor A. Reeves Jackson, of our collaborators' staff is one of the incorporators of the college.

IN CHICAGO, recently, the Health officers visited two churches on Sunday, and vaccinated the entire congregations. It is said the Health department meets with much opposition to vaccination from the ignorant residents of some of the north-side wards.

DR. JOSEPH J. HARTMAN, a promising young dentist of Richmond, Va., died in that city on the 17th of November last.

DR. J. B. BOUILLAUD, a French physician of celebrity, and an author and teacher of great distinction a quarter of a century ago, died on the 30th of October last.

PROF. D. WARREN BRICKELL, a well-known physician of New Orleans, La., died in that city on December 10th. Dr. Brickell had been ill for a long time and his death was not unexpected.

Dr. Chas. W. Tackenberg, of Cincinnati, O., died at Mentone, France, of Tuberculosis on December 4th, 1881. Dr. Tackenberg was only 25 years of age at the time of his death.

Dr. David Foulis, a young surgeon of distinction of Glasgow, Scotland, died recently of diphtheria, contracted during the performance of tracheotomy in two virulent cases of that disease.

Dr. Joshua Tucker, a prominent dentist of New England, died in Winchendon, Mass., on the 7th of November, aged eighty-one years. He was a member of the Mass. Med. Society, and an honorary member of the Odontological Society of Great Britain.

Dr. Robert Shelton Mackenzie, died in Philadelphia on the 21st of November, 1881, aged 74 years. Dr. Mackenzie was an Irishman by birth, and graduated in medicine in Dublin, but devoted himself to the literary profession instead of medicine. He was for many years Secretary and one of the Board of Trustees of the Philadelphia Dental School.

"If a doctor has the luck to find out a new malady, it is tied to his name like a tin kettle to a dog's tail, and he goes clattering down the highway of fame to posterity, with his æolo attachment following at his heels."—*Holmes.*

Legislative Logic. In Maine they have a law that no medical student shall be allowed to graduate in medicine who has not had regular practice in the dissecting room. Then a law was passed that no bodies, but those of executed criminals should be used as dissecting material, and then they abolished capital punishment and adjourned.

Dr. J. M. Fothergill, speaking of the character of attendants upon a sick person says :—"A pious widow, with dyspepsia and strong religious convictions, is a ghoul when illness is about. She sucks the life out of an invalid like a moral vampire. As life ebbs she is sustained, and when the invalid has passed the portals of another world she goes away edified, strengthened and encouraged in her murderous mission, fully prepared to extinguish the lives of any number of relatives if ill luck should prostrate them upon the sick bed."

